# An insulin-regulated arrestin domain protein controls hepatic glucagon action

**DOI:** 10.1016/j.jbc.2023.105045

**Published:** 2023-07-13

**Authors:** Sezin Dagdeviren, Megan F. Hoang, Mohsen Sarikhani, Vanessa Meier, Jake C. Benoit, Marinna C. Okawa, Veronika Y. Melnik, Elisabeth M. Ricci-Blair, Natalie Foot, Randall H. Friedline, Xiaodi Hu, Lauren A. Tauer, Arvind Srinivasan, Maxim B. Prigozhin, Sudha K. Shenoy, Sharad Kumar, Jason K. Kim, Richard T. Lee

**Affiliations:** 1 Department of Stem Cell and Regenerative Biology and the Harvard Stem Cell Institute, Harvard University, Cambridge, Massachusetts, USA; 2 Centre for Cancer Biology, University of South Australia, Adelaide, Australia; 3 Program in Molecular Medicine, University of Massachusetts Medical School, Worcester, Massachusetts, USA; 4 Department of Molecular and Cellular Biology, Harvard University, Cambridge, Massachusetts, USA; 5 John A. Paulson School of Engineering and Applied Sciences, Harvard University, Cambridge, Massachusetts, USA; 6 Division of Cardiology, Department of Medicine, Duke University Medical Center, Durham, North Carolina, USA; 7 Department of Cell Biology, Duke University Medical Center, Durham, North Carolina, USA; 8 Department of Medicine, Division of Endocrinology, Metabolism, and Diabetes, University of Massachusetts Medical School, Worcester, Massachusetts, USA

**Keywords:** arrestin, glucagon, ARRDC4, gluconeogenesis, insulin, glucagon resistance

## Abstract

Glucagon signaling is essential for maintaining normoglycemia in mammals. The arrestin fold superfamily of proteins controls the trafficking, turnover, and signaling of transmembrane receptors as well as other intracellular signaling functions. Further investigation is needed to understand the *in vivo* functions of the arrestin domain–containing 4 (ARRDC4) protein family member and whether it is involved in mammalian glucose metabolism. Here, we show that mice with a global deletion of the ARRDC4 protein have impaired glucagon responses and gluconeogenesis at a systemic and molecular level. Mice lacking ARRDC4 exhibited lower glucose levels after fasting and could not suppress gluconeogenesis at the refed state. We also show that ARRDC4 coimmunoprecipitates with the glucagon receptor, and ARRDC4 expression is suppressed by insulin. These results define ARRDC4 as a critical regulator of glucagon signaling and glucose homeostasis and reveal a novel intersection of insulin and glucagon pathways in the liver.

Insulin and glucagon act directly on the mammalian liver to control blood sugar levels. Insulin is a physiological inhibitor of glucagon release and function; however, it is incompletely understood at the molecular level how insulin exerts its effects on glucagon action (1). The relationship between insulin and glucagon signaling is more complex than a stochastic “on/off” switch, and the insulin:glucagon ratio forms a spectrum between the mobilization of stored nutrients to the biosynthesis of energy storage units (2). Although glucagon was discovered over a hundred years ago, glucagon resistance is a recently described concept (3), and glucagon resistance may be an important factor in the pathogenesis of type 2 diabetes (4, 5).

The arrestin fold superfamily of proteins integrates many intracellular signaling pathways, and some members control the inactivation, degradation, and signaling of G-protein–coupled receptors (GPCRs) (6). Several members of the branch of “alpha-arrestins” (to distinguish from beta/visual arrestins), including the arrestin domain–containing 3 (ARRDC3) protein and thioredoxin-interacting protein (TXNIP), are known to have important roles in mammalian metabolism (7, 8, 9, 10). ARRDC4 (also called DRH1) is a less well-studied member of the alpha-arrestin subfamily with some similarities to TXNIP and ARRDC3 (7), but there are very limited data on the *in vivo* functions of ARRDC4. ARRDC4 has been reported to interact with the neuronal precursor cell–expressed developmentally downregulated 4 (NEDD4) family of E3 ubiquitin ligases and thus may participate in receptor trafficking and ubiquitination (11, 12, 13). *Arrdc4* gene expression is controlled through carbohydrate-response elements by a MondoA-dependent mechanism (7, 14, 15), and high glucose levels induce *ARRDC4* expression in cultured human beta cells through MondoA, which is found to be important for insulin resistance and lipid metabolism (14, 16, 17). However, the role of ARRDC4 in mammalian glucose metabolism *in vivo* is unknown.

Here, we report an essential role of ARRDC4 in glucose metabolism *in vivo*. Our data show that in mice deficient in ARRDC4 (ARRDC4KO), hepatic glucose production in response to fasting and suppression of glucose production in response to feeding are both impaired. We show that *Arrdc4* null mice exhibit lower fasting glucose levels and have defective glucagon and gluconeogenesis responses. Furthermore, ARRDC4 interacts with the glucagon receptor–receptor complex, and the genetic deletion of *Arrdc4* causes an accumulation of glucagon receptors on the liver plasma membrane. We also demonstrate that *Arrdc4* in the liver is suppressed by insulin, and thus insulin can control hepatic glucagon signaling through ARRDC4. These findings reveal the ARRDC4 protein as a critical regulator of glucagon function and mammalian metabolism.

## Results

### ARRDC4KO mice demonstrate lower fasting glucose levels and impaired gluconeogenesis

**Figure 1:**
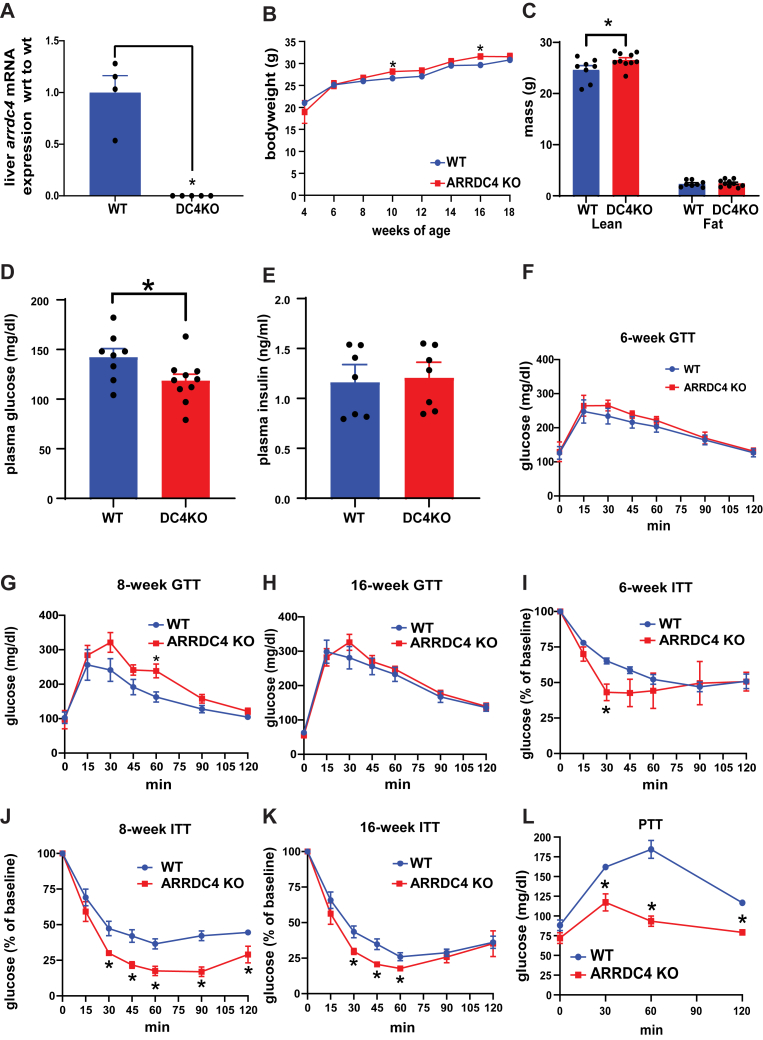
**ARRDC4KO mice exhibit lower fasting glucose levels and cannot restore normal glucose levels following insulin or pyruvate stimulation.***A*, liver *Arrdc4* mRNA expressions of WT and ARRDC4KO/DC4KO mice (n = 4–5). *B*, bodyweights of male WT and ARRDC4KO mice (n = 5–7) between 4 and 18 weeks of age. *C*, lean and fat masses of 16-week-old male WT and ARRDC4KO mice measured using 1H-MRS (n = 8–10). *D*, plasma glucose values of 16-h-fasted 16-week-old male WT and ARRDC4KO mice (n = 8–10). *E*, plasma insulin levels of 5-h-fasted male WT and ARRDC4KO mice (n = 7). *F*–*K*, 16 h fasted glucose (2 g/kg) (GTT) and 5-h-fasted insulin (ITT) (0.75 IU/kg) tolerance tests in 6-, 8-, and 16-week-old male ARRDC4KO and WT mice (n = 5–7). *L*, 16-h-fasted pyruvate tolerance tests (PTTs; 2 g/kg) in 12-week-old male ARRDC4KO and WT mice (n = 7) (∗*p* < 0.05, values are mean ± SEM, unpaired two-tailed Student’s *t* test). ARRDC, arrestin domain–containing protein.

We studied the ARRDC4KO mouse to understand the protein’s metabolic roles in depth. We confirmed the ablation of *Arrdc4* in ARRDC4KO mice livers by quantitative PCR (Fig. 1*A*). We measured the body weights of ARRDC4KO and WT mice on a regular chow diet. While the weights of ARRDC4KO mice were elevated at some time points compared with controls, we did not observe a significant difference in weights by the end of 18 weeks (Fig. 1*B*). We measured the total lean mass and fat mass of the mice using proton magnetic resonance spectroscopy to compare their adiposity. While ARRDC4KO mice had slightly higher lean masses, there was no difference in fat masses compared with controls (Fig. 1*C*). ARRDC4KO mice showed significantly lower glucose levels following an overnight fast, exhibiting a low fasting glucose phenotype (Fig. 1*D*), and this was not because of a difference in plasma insulin levels (Fig. 1*E*).

Glucose tolerance tests (GTTs) performed at 6 and 16 weeks of age did not demonstrate a difference in glucose tolerance of ARRDC4KO compared with WT mice. WT mice had higher glucose excursion at week 8 (Fig. 1, *F*–*H*). However, ARRDC4KO mice exhibited decreased glucose levels during insulin tolerance tests as plasma glucose levels fell more robustly than WT mice after insulin injection and did not recover as rapidly as WT mice at 8 and 16 weeks of age (Fig. 1, *I*–*K*). Next, we performed pyruvate tolerance tests (PTTs) and found that ARRDC4KO mice showed impaired gluconeogenesis following pyruvate injection compared with WT mice (Fig. 1*L*). These findings suggested that ARRDC4KO mice have defective hepatic glucose production in the fasting state.

### Glucagon-stimulated gluconeogenesis is defective in ARRDC4KO mice

**Figure 2:**
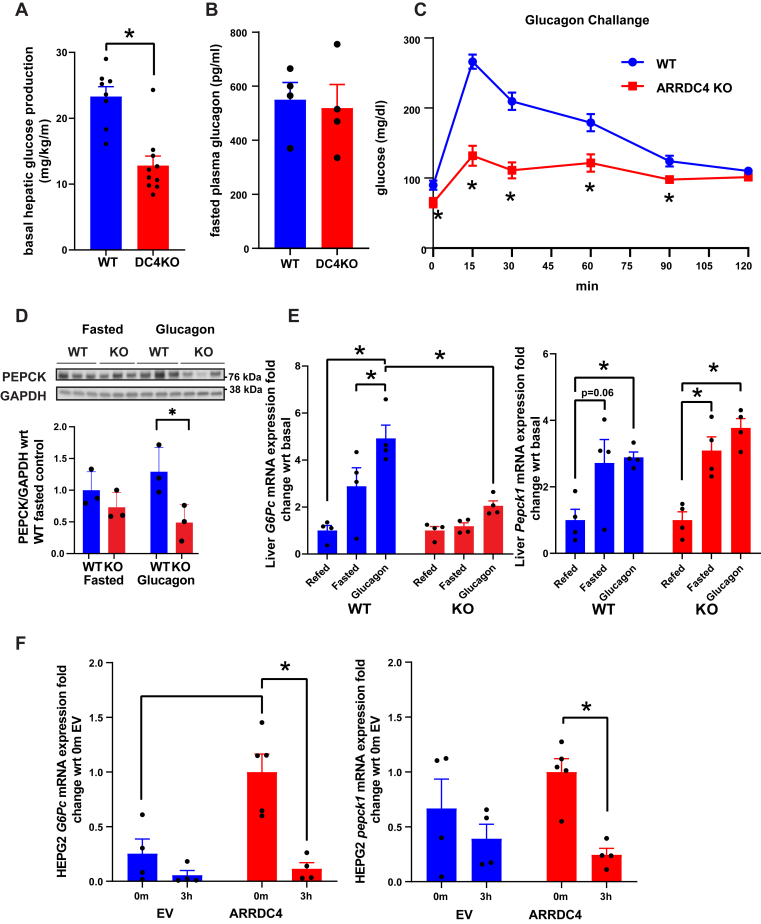
**Loss of ARRDC4 impairs glucagon-stimulated glucose production because of glucagon resistance**. *A*, basal hepatic glucose production levels of 16-week-old ARRDC4KO and WT mice (n = 8–10). *B*, plasma glucagon levels of 16-h-fasted male ARRDC4KO and WT mice (n = 8–10). *C*, 16-h-fasted glucagon challenge test (20 μg/kg) of 12-week-old ARRDC4KO and WT mice (n = 7). *D*, Western analyses for PEPCK proteins in WT and ARRDC4KO mice livers after 16 h fasting and 16 h fasting + 50 μg/kg glucagon stimulation (n = 3). *E*, liver *G6pc* and *Pepck1* mRNA expressions following 16 h fasting,16 h fasting + 6 h refeeding, or 16 h fasting + glucagon stimulation in ARRDC4KO and WT mice that are normalized to refed basal levels in ARRDC4KO and WT mice, respectively (n = 4). *F*, *G6Pc* and *Pepck1* mRNA levels in HEPG2 cells that are transfected with ARRDC4 or empty vector and stimulated with insulin for 3 h (n = 5) (∗*p* < 0.05, values are mean ± SEM, two-way ANOVA and unpaired two-tailed Student’s *t* test). ARRDC, arrestin domain–containing protein; PEPCK, phosphoenolpyruvate carboxykinase, G6Pc, glucose-6-phosphatase.

To investigate whole-body glucose metabolism and liver function in ARRDC4KO mice, a 2 h hyperinsulinemic–euglycemic clamp was performed in awake mice (18). Basal hepatic glucose production was decreased by ∼50% in ARRDC4KO mice compared with WT mice, consistent with the low fasting glucose phenotype (Fig. 2*A*). Some beta-arrestins are known to regulate glucagon receptor turnover (19, 20, 21); thus, to understand if the gluconeogenesis defect in ARRDC4KO animals was due to glucagon resistance, we conducted glucagon challenge tests. ARRDC4KO mice showed significantly lower plasma glucose levels following glucagon injection despite similar endogenous glucagon levels, revealing glucagon resistance in ARRDC4KO mice (Fig. 2, *B* and *C*). To study the decline of gluconeogenesis at a molecular level, we assessed the major gluconeogenesis enzymes: phosphoenolpyruvate carboxykinase (PEPCK) and glucose 6-phosphatase (G6Pase). Livers of ARRDC4KO mice showed decreased PEPCK protein levels compared with WT mice only in the glucagon-treated state (Figs. 2*D* and S1*A*). We also measured *G6pc* mRNA levels in refed, fasted, and glucagon-stimulated livers of ARRDC4KO and WT mice. *G6pc* mRNA expression levels in ARRDC4KO and WT livers in refed (basal), fasted, and glucagon-stimulated states were normalized to basal levels of the respective group. We found that *G6pc* mRNA levels were increased robustly upon glucagon induction in WT mice; however, this was not observed in ARRDC4KO mice (Fig. 2*E*). At the protein level, G6Pase expressions were higher in the fasted state compared with the refed state in both groups (Fig. S1*A*). *Pepck1* mRNA levels increased with fasting/glucagon treatment in the ARRDC4KO group similar to the WT group (Fig. 2*E*). These data showed that ARRDC4KO mice do not respond normally to glucagon stimulation.

We also overexpressed hARRDC4 protein in HEPG2 cells to detect how it affects insulin-stimulated suppression of *G6PC* and *PEPCK1*. *G6PC* mRNA expression was already higher in cells overexpressing ARRDC4 at 0 min, agreeing with the role of ARRDC4 in gluconeogenesis (Fig. 2*F*). We saw that ARRDC4 overexpressing insulin-stimulated HEPG2 cells suppressed *G6PC* and *PEPCK1* expression much more efficiently than controls, supporting the role of ARRDC4 in insulin control of gluconeogenesis suppression at the transcriptional level (Fig. 2*F*). We did not detect this difference at the protein level (Fig. S1*B*).

### ARRDC4 ablation decreases cAMP and cAMP response element–binding protein levels in the liver

**Figure 3:**
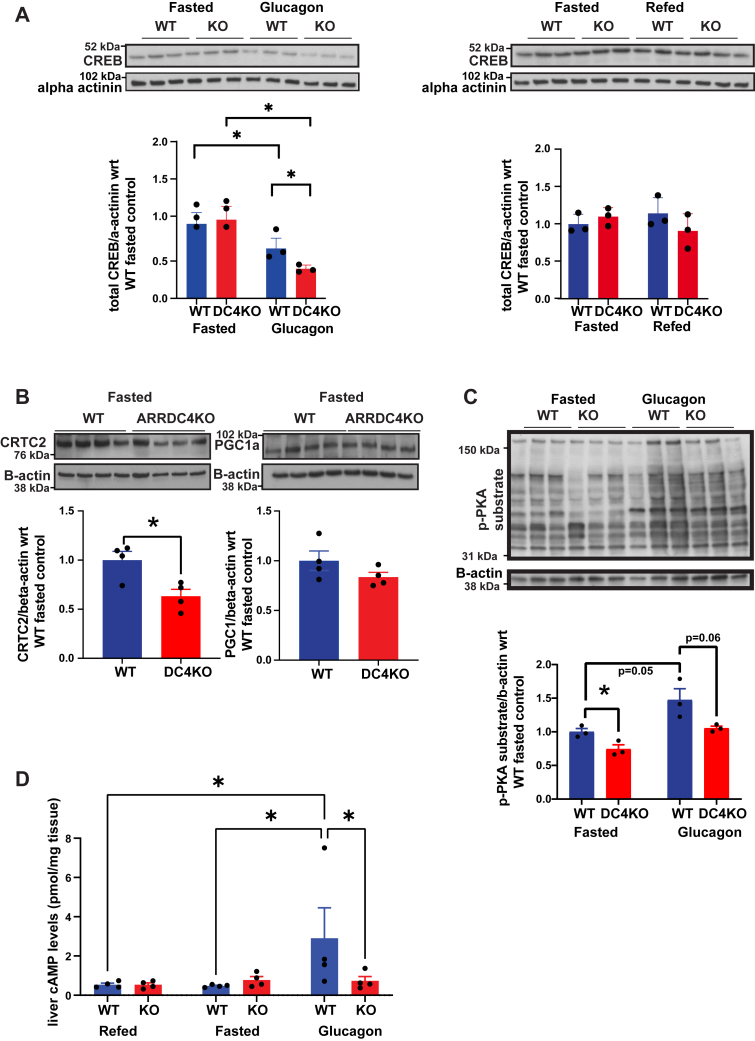
**Loss of ARRDC4 results in depletion of CREB protein and cAMP levels.***A*, total hepatic CREB levels following 16 h fasting and 6 h refeeding, 16 h fasting or 16 h fasting + glucagon stimulation (n = 3). *B*, total hepatic CRTC2 and PGC-1α protein levels following 16 h fasting. *C*, hepatic P-PKA substrate levels following 16 h fasting or 16 h fasting + glucagon stimulation (n = 3). *D*, hepatic cAMP levels following 16 h fasting and 6 h refeeding, 16 h fasting or 16 h fasting + glucagon stimulation in WT and ARRDC4KO mice (n = 4) (∗*p* < 0.05, values are mean ± SEM, two-way ANOVA and unpaired two-tailed Student’s *t* test). ARRDC, arrestin domain–containing protein; CREB, cAMP response element–binding protein; CRTC2, CREB-regulated transcription coactivator 2; PGC-1α, peroxisome proliferator–activated receptor gamma coactivator alpha.

cAMP response element–binding protein (CREB) is induced by glucagon and drives *PEPCK1* and *G6PC* gene transcription by binding to cAMP-response elements to enhance gene expression with a subsequent increase in hepatic gluconeogenesis (22). CREB plays a role in maintaining basal PEPCK protein expression in the absence of insulin (23). CREB protein levels were decreased in ARRDC4KO livers in the glucagon-stimulated state (Fig. 3*A*), but CREB phosphorylation was not altered (Fig. S1*C*). The difference in CREB protein levels was not observed at the total mRNA level, suggesting that this could be a post-translational effect. CREB-regulated transcription coactivator 2 (CRTC2) is a coactivator of the CREB protein and a major mediator of the cAMP signaling pathway in the liver to regulate gluconeogenesis (24). Peroxisome proliferator–activated receptor gamma coactivator alpha (PGC-1α) is also an important regulator of gluconeogenesis (25). The CREB–CRTC2 complex promotes the transcription of major gluconeogenic markers, such as PGC-1α, PEPCK, and G6PC, to increase gluconeogenesis (26). For this reason, we also checked the protein levels in WT and ARRDC4KO mouse livers at the overnight fasted state. We detected a significant decrease in CRTC2 protein levels in ARRDC4KO livers compared with WT livers agreeing with the decreased gluconeogenesis, CREB levels, and PEPCK levels (Fig. 3*B*). The difference in PGC-1α protein levels did not reach significance (Fig. 3*B*).

We also assessed the glucagon signaling pathway in ARRDC4KO and WT livers. PKA activity levels increased in the glucagon-treated groups compared with the basal refed groups, but we did not detect a significant difference between WT and ARRDC4KO mice (Fig. S1*D*). PKA substrate phosphorylation levels were higher in the glucagon state than the fasting state in WT mice, and the ARRDC4KO group’s phosphorylation levels were lower than the WT group’s levels, implying a decrease in PKA phosphorylation with the absence of ARRDC4 (Fig. 3*C*). Interestingly, cAMP levels also increased after glucagon stimulation in WT mice but not in ARRDC4KO mice (Fig. 3*D*). These experiments demonstrate that some key proteins associated with the glucagon signaling pathway are reduced in the ARRDC4KO mouse.

### ARRDC4 interacts and colocalizes with glucagon receptors in HEPG2 cells and controls its localization in liver tissue

**Figure 4:**
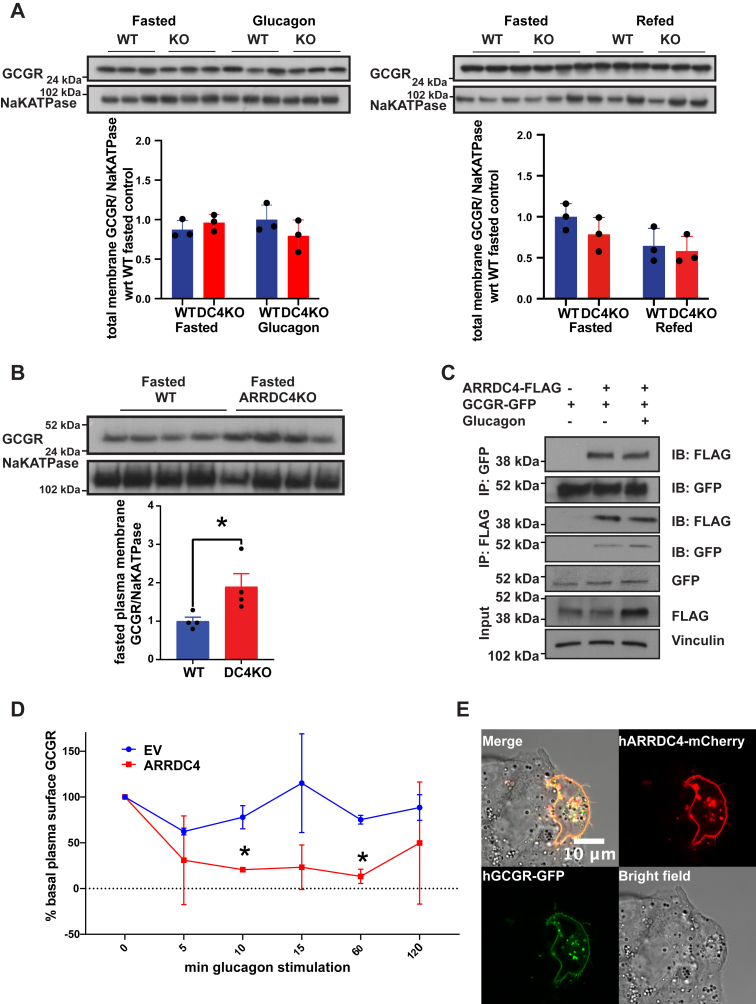
**ARRDC4 interacts and colocalizes with the glucagon receptor and is responsible for glucagon receptor localization.***A*, liver total membrane fraction glucagon receptor protein levels in 16-h-fasted 6-h-refed, 16-h-fasted, and 16-h-fasted + glucagon-stimulated WT and ARRDC4KO mice (n = 3). *B*, liver plasma membrane fraction glucagon receptor protein levels in 16-h-fasted and WT and ARRDC4KO mice (n = 4). *C*, coimmunoprecipitation assay of human HEPG2 liver cells transiently overexpressing FLAG-tagged hARRDC4 + GFP-tagged hGCGR or GFP-tagged hGCGR + EV with or without the presence of glucagon stimulation. Lysates were both pulled down with FLAG and blotted with GFP antibodies or pulled down with GFP and blotted with FLAG antibodies. *D*, plasma surface glucagon receptor levels compared with basal levels in EV or hARRDC4 + human glucagon receptor transfected HEK293 cells over time-course glucagon stimulation. *E*, representative confocal images of HEPG2 cells transfected with mCherry-hARRDC4 and GFP-hGCGR plasmids. (∗*p* < 0.05, values are mean ± SEM, unpaired two-tailed Student’s *t* test). ARRDC, arrestin domain–containing protein; EV, empty vector; HEK293, human embryonic kidney 293 cell line.

Glucagon receptors are GPCRs. Beta arrestin 1 and 2 (27, 28), ARRDC3, and ARRDC4 (12, 29) can regulate GPCR turnover and degradation (7, 30). Furthermore, ARRDC4 is known to interact with the NEDD4 family of ubiquitin ligases to ubiquitinate membrane receptors for breakdown (11, 12, 13). To determine whether ARRDC4 regulates glucagon receptor turnover and trafficking, we isolated total membrane and plasma membrane fractions from livers of ARRDC4KO mice. We did not observe a difference in glucagon receptor levels between total membrane fractions of ARRDC4KO and WT livers (Fig. 4*A*). However, plasma membrane fractions of ARRDC4KO livers had more glucagon receptors than the WT group, suggesting that glucagon receptors are trapped at the plasma membrane because of a lack of ARRDC4-facilitated turnover of membrane proteins (Fig. 4*B*). Next, we sought to understand if ARRDC4 can interact with glucagon receptors. We performed coimmunoprecipitation experiments in HEPG2 human liver cancer cell lines that were transfected with human glucagon receptor-GFP + human ARRDC4-FLAG or glucagon receptor + empty vector controls and treated with glucagon or PBS. The cell lysates were pulled down with FLAG or GFP antibodies and blotted for GFP and FLAG antibodies. ARRDC4 coimmunoprecipitated with glucagon receptors in HEPG2 cells regardless of the glucagon-binding state (Fig. 4*C*). We also tried to confirm that ARRDC4 is helping to transport glucagon receptors to intracellular membranes from the cell surface. We stimulated human embryonic kidney 293 (HEK293) cells transfected with either empty vector or hARRDC4 + hGlucagon receptor with glucagon for 5, 10, 15, 60, or 120 min. The results showed that surface glucagon receptors in control cells decreased slightly at first but reached back to basal levels after 15 min. However, in the cells transfected with hARRDC4, the plasma glucagon receptors decreased significantly and stayed low for a long time, suggesting they were forced to internalize to a greater extent (Fig. 4*D*). We also expressed hARRDC4-mCherry and hGCGR-GFP plasmids in HEPG2 cells to detect the respective protein’s localization. hARRDC4 and hGCGR proteins were colocalized on human liver cell membranes according to confocal images (Fig. 4*E*). These results show that ARRDC4 immunoprecipitates with the glucagon receptor and colocalizes with the glucagon receptor in HEPG2 cells. In the absence of ARRDC4, glucagon receptors accumulate in the plasma membrane, and by overexpression of ARRDC4, they might be directed toward internalization.

### ARRDC4KO mice fail to suppress glucose production

**Figure 5:**
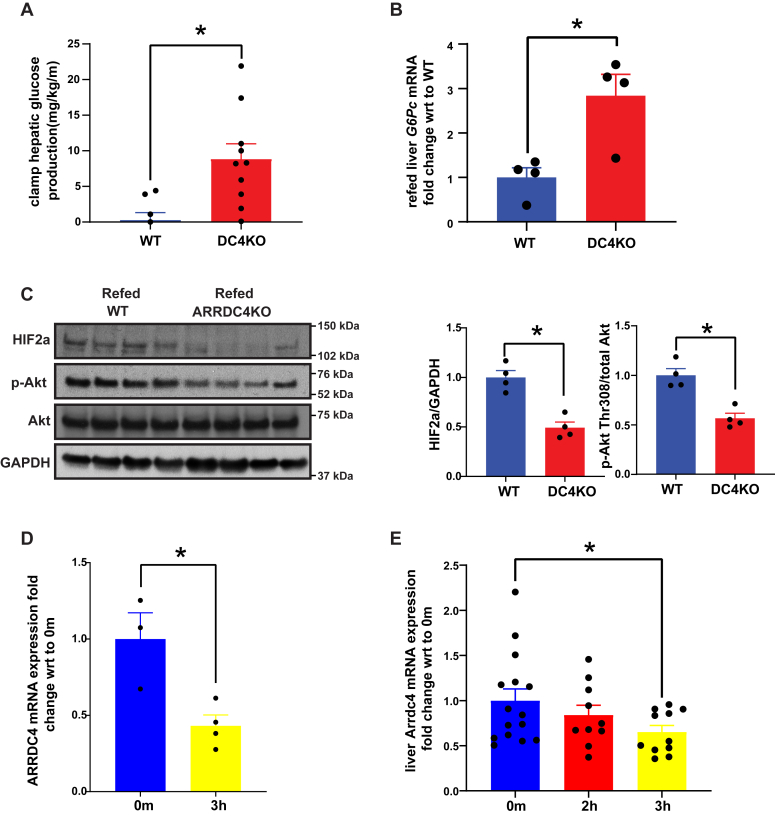
**ARRDC4KO mice fail to suppress hepatic glucose production.***A*, clamp hepatic glucose production in WT and ARRDC4KO mice during hyperinsulinemic–euglycemic clamps (n = 8–10). *B*, *G6pc* mRNA expression in livers of 16-h-fasted + 6-h-refed WT and ARRDC4KO mice that are normalized to the WT group's expression (n = 4). *C*, HIF2 alpha protein and Akt Thr308 phosphorylation levels in livers of WT and ARRDC4KO mice under 16-h-fasted + 6-h-refed conditions (n = 4). *D*, *ARRDC4* mRNA expression in HEPG2 cells stimulated with 10 nm insulin (n = 4) for 3 h. *E*, liver *Arrdc4* mRNA expressions of mice 2 h or 3 h after being injected i.p. with insulin (2 IU, n = 10–15) (∗*p* < 0.05, values are mean ± SEM, unpaired two-tailed Student’s *t* test used, graphics created by BioRender.com). ARRDC, arrestin domain–containing protein; HIF2, hypoxia-inducible factor 2.

Alterations in hepatic glucose metabolism in ARRDC4KO mice were not limited to fasting conditions. In ARRDC4KO mice, hyperinsulinemic–euglycemic clamp experiments showed that hepatic glucose production during the insulin-stimulated state was significantly increased compared with WT mice, indicating hepatic insulin resistance–related insufficient suppression of glucose output in these mice (Fig. 5*A*). These data imply that ARRDC4 is not only just essential for gluconeogenesis in the fasting state but also plays a critical role in suppressing gluconeogenesis in the insulin-stimulated state. We also observed increased *G6pc* mRNA expression levels in refed ARRDC4KO mice livers compared with WT levels when *G6pc* expression in WT and ARRDC4KO livers in the refed state was normalized to the WT group's expression (Fig. 5*B*). We did not see this trend for *Pepck1* expression (Fig. S1*E*). PKA substrate phosphorylation levels were decreased in WT mice by refeeding, but the difference between WT and ARRDC4KO groups was not significant (Fig. S1*F*).

Hypoxia-inducible factor 2 (HIF2) alpha has recently been shown to be upregulated after refeeding and to inhibit the glucagon signaling pathway (31, 32). Phosphatidylinositol-dependent kinase 1/PI3K activation after insulin stimulation induced by refeeding also triggers AKT-Thr308 phosphorylation that provides the phosphate for phosphorylation/nuclear exclusion of Forkhead box protein O1 to terminate gluconeogenic gene expression (33). We found that HIF2 alpha protein levels, as well as AKT phosphorylation, were decreased in the livers of ARRDC4KO mice in the refed state (Fig. 5*C*). We measured liver and plasma lipid levels, and glucagon resistance did not affect lipid metabolism adversely (Fig. S2, *A*–*C*). These findings show that ARRDC4 plays a critical role in suppressing gluconeogenesis.

### Insulin suppresses ARRDC4 expression

Given the essential role of ARRDC4 in regulating glucagon function, we explored the association between ARRDC4 and insulin action. Since *Arrdc4* is a glucose-induced gene (15), we investigated the expression of *ARRDC4* mRNA in insulin-induced HEPG2 cells. We observed that insulin decreased *ARRDC4* mRNA expression in HEPG2 cells (Fig. 5*D*). We also noted a similar decline in the livers of insulin-treated mice (Fig. 5*E*).

### ARRDC4KO mice also show decreased insulin sensitivity

**Figure 6:**
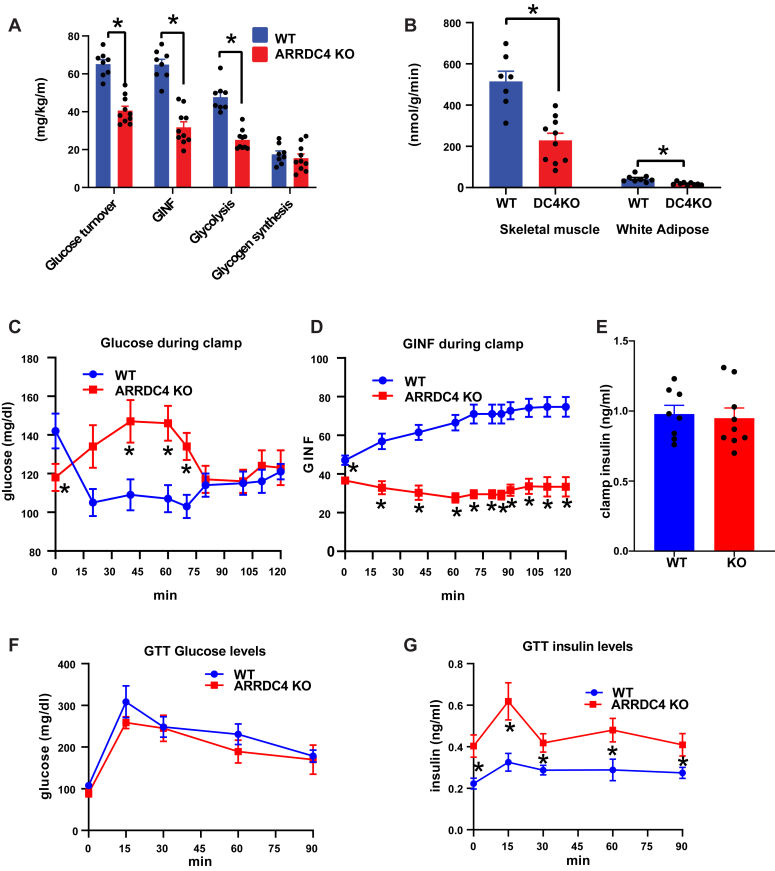
**ARRDC4KO mice show decreased insulin sensitivity**. *A*, whole-body glucose turnover, whole-body glucose infusion rate, whole-body glycolysis, and whole-body glycogen and lipid synthesis rates during hyperinsulinemic–euglycemic clamps in WT and ARRDC4KO mice (n = 8–10). *B*, insulin-stimulated glucose uptake in skeletal muscle (gastrocnemius) and white adipose tissue (epidydimal) of WT and ARRDC4KO mice (n = 7–10). *C* and *D*, glucose and glucose infusion rate levels during hyperinsulinemic–euglycemic clamps in WT and ARRDC4KO mice (n = 8–10). *E*, plasma insulin levels at the end of hyperinsulinemic–euglycemic clamps in WT and ARRDC4KO mice (n = 8–9). *F* and *G*, plasma glucose and insulin levels during GTT experiment (16-h-fasted, 2 g/kg) in WT and ARRDC4KO mice (n = 7) (∗*p* < 0.05, values are mean ± SEM, unpaired two-tailed Student’s *t* test used). ARRDC, arrestin domain–containing protein; GTT, glucose tolerance test.

Hyperinsulinemic–euglycemic clamp experiments showed that ARRDC4-deficient mice have reduced insulin sensitivity as well as glucagon resistance. Whole-body glucose turnover, glycolysis, and glucose infusion rates were reduced in ARRDC4KO mice compared with WT mice (Fig. 6*A*). This was mostly because of the changes in peripheral insulin action as insulin-stimulated glucose uptake rates in skeletal muscle and white adipose tissue were significantly decreased in ARRDC4KO mice compared with WT mice (Fig. 6*B*). Postclamp insulin levels and steady-state glucose levels during the last half an hour of the clamp were similar between the groups as expected from a functional clamp experiment, but continuously lower glucose infusion levels during clamp revealed decreased insulin sensitivity in the knockout mice (Fig. 6, *C*–*E*). We did not observe significant changes in basal and postclamp levels of phospho-Akt or total Akt between groups in the liver (Fig. S3, *A* and *B*). We also performed a GTT time-course experiment to measure insulin levels during the GTT. The data showed that ARRDC4KO mice had higher insulin levels during the glucose stimulation compared with WT mice, consistent with the decreased insulin sensitivity phenotype (Fig. 6, *F* and *G*).

### Glycogen, ketone, lactate, and nonesterified fatty acid metabolism are altered in ARRDC4KO mice

**Figure 7:**
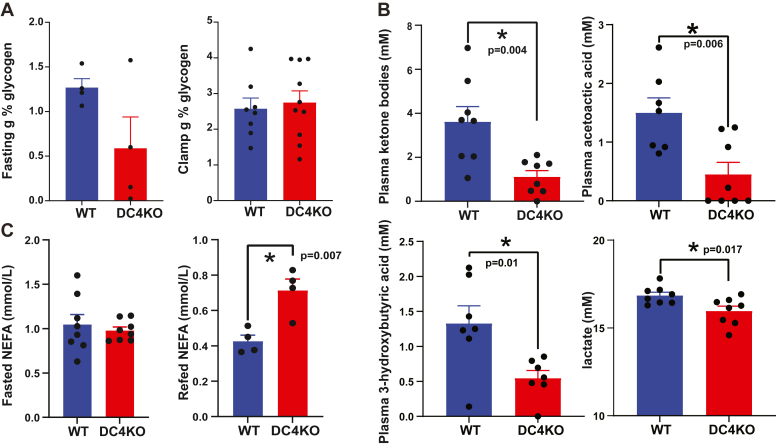
**Glycogen, ketone, lactate, and NEFA metabolism are altered in ARRDC4KO mice.***A*, 16-h fasting (n = 4) and postclamp (n = 8–10) total current glycogen levels in livers of WT and ARRDC4KO mice. *B*, plasma ketone bodies, acetoacetic acid, 3-hydoxybutyric acid, and lactate levels in 16-h-fasted WT and ARRDC4KO mice (n = 7–8). *C*, plasma NEFA levels in 16-h-fasted (n = 8) and 16-h-fasted + 6-h-refed (n = 4) WT and ARRDC4KO mice (*p* < 0.05, values are mean ± SEM, and unpaired two-tailed Student”s *t* test). ARRDC, arrestin domain–containing protein; NEFA, nonesterified fatty acid.

We measured fasting and postclamp glycogen levels in the livers of WT and ARRDC4KO mice. Although there was no significant decrease, fasting glycogen levels were slightly lower in the ARRDC4KO mice (Fig. 7*A*). Therefore, we wanted to assess the glycogen metabolism signaling pathway in liver tissues. The inactive phosphorylated state of glycogen synthase was slightly elevated in ARRDC4KO mice in fasted livers, agreeing with slightly lower levels of glycogen; however, these differences did not reach significance. We did not observe a difference in total GS, phosphor, and total GS kinase, and phospho and total PYGL protein levels (Fig. S3, *C*–*E*).

Ketogenesis is negatively and positively regulated by insulin and glucagon, respectively (34). Since we found glucagon resistance and insulin resistance phenotypes in the absence of ARRDC4, we measured circulating levels of ketone bodies in the ARRDC4KO mice. The fasting (16 h) plasma ketone body levels, composed of acetoacetic and 3-hydroxybutyric acid, were decreased significantly in ARRDC4KO mice (Fig. 7*B*). Plasma lactate levels were also significantly reduced in ARRDC4KO mice in the 16 h fasted state compared with WT controls (Fig. 7*B*).

Prolonged increase of plasma nonesterified fatty acid (NEFA) levels reduces insulin-stimulated glucose utilization in peripheral tissues, and atypically high levels of NEFA are thought to play an essential role in the development of type 2 diabetes (35). The reduction of insulin-stimulated suppression of plasma NEFA levels in the postprandial state is one of the earliest metabolic dysfunctions detected in patients at high risk of diabetes (36). Therefore, we also checked the plasma NEFA levels in 16 h fasted ARRDC4KO mice and compared them with 16 h fasted + 6 h refed mice plasma levels. Refeeding caused a decrease in NEFA levels from ∼1 to a ∼0.4 mmol/l range in WT mice (Fig. 7*C*). However, NEFA levels were significantly higher in ARRDC4KO mice compared with WT mice after refeeding (Fig. 7*C*), likely pointing to a decreased suppression of NEFA by insulin, which may result in decreased insulin action in the long run.

## Discussion

**Figure 8:**
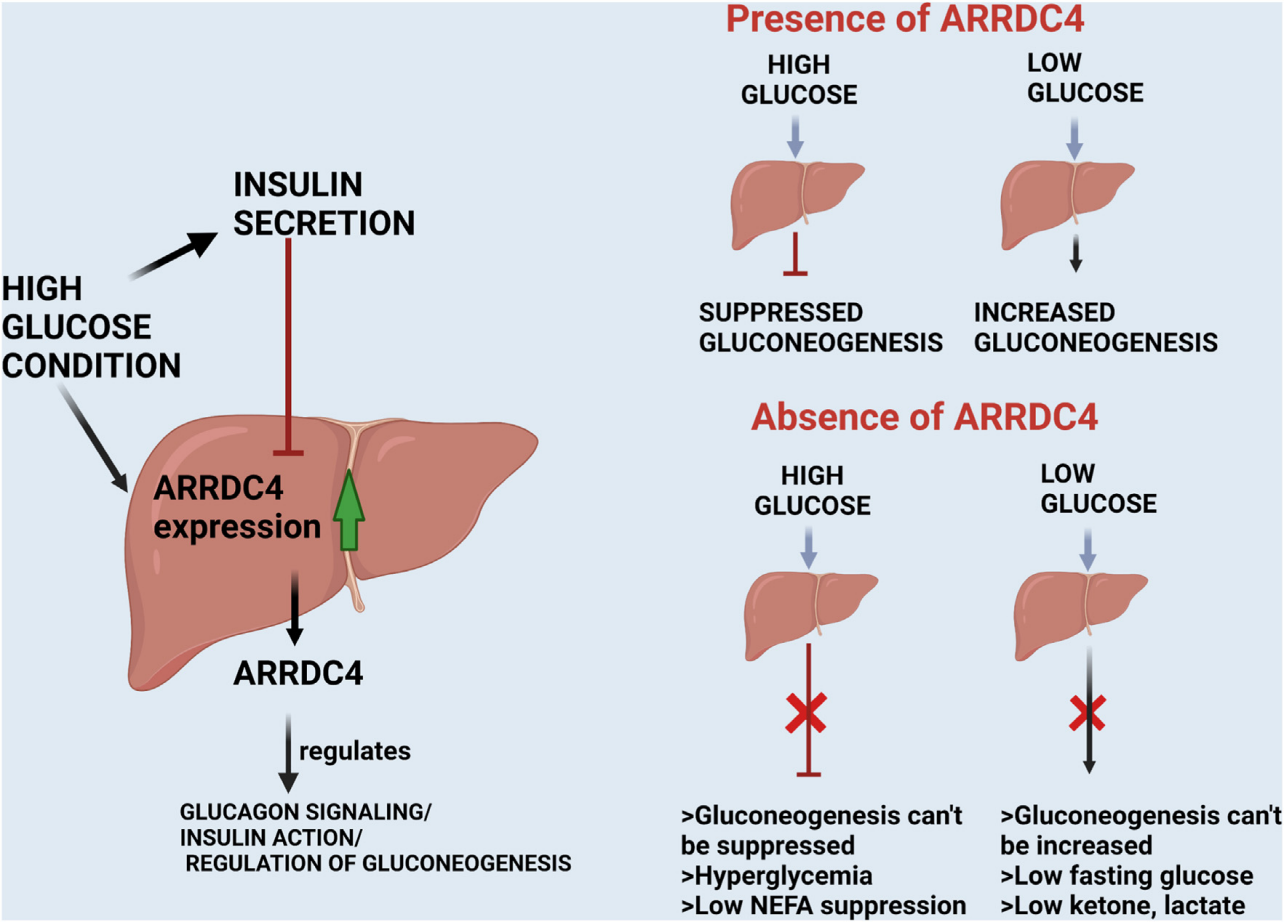
**Schematic representation of ARRDC4 roles in metabolism.** Under normal conditions, ARRDC4 is induced by glucose and repressed by insulin to control gluconeogenesis in low and high glucose states. The absence of ARRDC4 disturbs the feedback from the blood glucose levels to regulate hepatic glucose output. Without ARRDC4, the gluconeogenesis pathway is not activated sufficiently, and low glucose levels occur in the fasted state. Even though glucose and insulin are present, gluconeogenesis is not suppressed in the absence of ARRDC4 (graphics created by BioRender.com). ARRDC, arrestin domain–containing protein.

Insulin and glucagon participate in a dynamic network to maintain blood euglycemic levels (37). Here, we define an important *in vivo* role for the ARRDC4 protein in the regulation of glucagon-mediated glucose production. We demonstrate that *Arrdc4* gene expression is suppressed by insulin in the liver and cultured cells. ARRDC4 regulates gluconeogenesis through glucagon signaling. Our findings agree with Batista *et al*. (38), who showed that *Arrdc4* is one of the downregulated genes in the liver during a physiological insulin clamp. We also demonstrate that ARRDC4 has dual roles in maintaining euglycemia in both fasted and refed conditions. These ARRDC4KO mice also showed insulin resistance along with glucagon resistance, both of which contribute to defective glucose homeostasis in diabetic states. The GTT–insulin data showed that ARRDC4KO mice had higher insulin levels during the glucose stimulation compared with WT mice. The data suggest that there is increased insulin secretion following glucose injection, and this may be due to loss of glucagon action as well, which would result in impaired feedback inhibition of insulin secretion by the beta cells. These experiments define ARRDC4 as a critical protein in the glucagon response and glucose production during both fasted, refed, and insulin-stimulated states and reveal a previously unknown mechanism by which insulin and glucose can control hepatic glucose metabolism (15) (Fig. 8).

ARRDC4KO mice have previously been shown to have defective extracellular vesicle release and sperm maturation (11, 39, 40). Here, we further show that ARRDC4KO mice exhibit low fasting glucose levels and cannot generate sufficient glucose following overnight fasting. During pyruvate and glucagon challenge tests, we found that this defect was due to a lack of response to glucagon stimulation even though the mice had similar levels of endogenous glucagon. Glucagon resistance is a recent concept and refers to the impairment of glucagon-induced signaling and metabolism (3, 41, 42). Here, we reveal ARRDC4 as a potential intracellular factor that plays a role in hepatic glucagon resistance. Our molecular data also agree with impaired gluconeogenesis in the absence of ARRDC4. PEPCK and *G6pc* were downregulated in ARRDC4KO mice, in addition to cAMP, CREB, and CRTC2, which are induced directly by glucagon (43). Although CREB and its effectors entail cAMP-mediated signaling, our results do not define if ARRDC4 modulates GCGR-Gs coupling or Gs activation, which would be an interesting subject for future investigation.

Beta and visual arrestin proteins regulate GPCR turnover (44), and alpha-arrestin proteins may also serve as adaptors for GPCR degradation (30). ARRDC3 was recently shown to interact with the GPCR beta-2 adrenergic receptor (29, 45). ARRDC4 interacts with E3 ubiquitin ligases, such as NEDD4, tripartite motif 65, and itchy E3 ubiquitin protein ligase (11, 12, 46). The NEDD4 family can play a crucial role in the regulation of several membrane receptors and endocytic machinery elements (47). To investigate whether the gluconeogenesis defect in ARRDC4KO mice was due to a defect in recycling glucagon receptors, we measured glucagon receptor levels in the plasma membrane and total membrane fractions of ARRDC4KO and WT livers. If ARRDC4 functioned to ubiquitinate glucagon receptors and target them for internalization, we would expect an accumulation of the glucagon receptors in ARRDC4KO liver plasma membranes; this was indeed the case. These results suggest that the lack of ARRDC4 caused an accumulation of glucagon receptors in the plasma membrane, preventing them from entering the receptor turnover cycle. Internalization experiments also showed that overexpression of ARRDC4 increased the internalization of the glucagon receptors from the plasma membrane into internal membranes. It is plausible that more receptors in the membrane would increase signaling; however, recent findings show that basal status of ubiquitination and frequent deubiquitination are important for responsiveness of some GPCRs including the glucagon receptor (48, 49). Emerging findings now support the concept that GPCR endocytosis is important for continuing signaling (50, 51, 52) and prolonged function (53). Also, to show the ARRDC4–glucagon receptor interaction at the molecular level, we performed coimmunoprecipitation experiments in the human HEPG2 cell line and observed that ARRDC4 and the glucagon receptor interact regardless of glucagon treatment. We currently do not know if this interaction is direct or through a super complex as the lysates were not purified. The interaction might be taking place through other proteins. We were also able to show that human ARRDC4 and GCGR proteins colocalized on HEPG2 human liver cell membranes. It remains to be determined whether ARRDC4 plays a role in the ubiquitination of the glucagon receptor by interacting with a specific ubiquitin ligase.

We currently do not know if the sole function of the ARRDC4 protein in hepatocytes is to facilitate glucagon receptor internalization. Alternative hypotheses are also possible, such as during the refeeding state, ARRDC4 might be affecting signaling in addition to enhancing sustained G protein–dependent cAMP production in endosomes. It is plausible that alpha-arrestins can have analogous functions to beta-arrestins, which play broad regulatory roles in mammalian cellular physiology/signaling and have numerous binding partners (54). Researchers have identified hundreds of binding partners for beta-arrestins in metabolic and signaling pathways, which they call the “beta-arrestinome” (55). This arrestinome is known to include a range of kinase proteins, most of which are involved in promoting arrestin-dependent signaling. Beta-arrestins also interact with clathrin, which promotes receptor internalization and endocytosis (56). Desensitized receptor–arrestin complexes are targeted for arrestin-mediated sequestration *via* clathrin-coated pits and are either recycled to the cell surface or sent for degradation (57). Beta-arrestins also recruit Src tyrosine kinases and control binding and receptor internalization that terminate G protein activation and are important for the initiation of mitogenic signals from the GPCR (57). Thus, it is possible that alpha arrestins are regulating GPCR signaling *via* these similar pathways.

Our data also revealed that the role of ARRDC4 in maintaining glucose levels is not confined to fasting conditions. Mice that lacked ARRDC4 also had impaired suppression of hepatic glucose production in hyperinsulinemic–euglycemic clamps during both normoglycemia and hyperinsulinemia. ARRDC4 deficiency seems to exacerbate both hyperglycemia and low glucose levels by failing to regulate gluconeogenesis. Recently, the HIF2 alpha/extracellular signal–regulated kinase pathway has been found to be essential for inhibiting cAMP-elevated gluconeogenic gene expression (31, 32). The insulin/p-Akt pathway also increases Forkhead box protein O1 phosphorylation to prevent FOXO from entering the nucleus to drive gluconeogenic gene transcription (58). Here, we observed that HIF2 alpha protein levels, as well as Akt phosphorylation, were decreased in ARRDC4KO mice upon refeeding. Overexpression of hARRDC4 in HEPG2 cells also increased insulin’s ability to suppress *PEPCK1* and *G6PC* genes, highlighting the importance of ARRDC4 in gluconeogenesis in human cells as well.

We also detected defects in glycogen, ketone, and NEFA metabolism in ARRDC4KO mice. Glycogen phosphorylase has a direct effect on blood glucose levels through the glycogenolysis pathway. The decrease in glycogen phosphorylase activation by glucagon through the cAMP pathway in ARRDC4KO mice in the fasting and glucagon-stimulated states could explain the defect in generating enough glucose during the fasting state in these mice. In addition to controlling glucose metabolism, glucagon is also known to induce ketosis during fasting (59). Consistent with our decreased glucagon action phenotype, plasma ketone bodies were found to be lower in ARRDC4KO mice compared with controls. Some studies have suggested that glucagon increases lactate levels (60, 61, 62). We found lower levels of plasma lactate in ARRDC4KO mice compared with controls, which might be related to lower glucagon action. In summary, the opposite phenotype of lactic acidosis and ketoacidosis that occurs during diabetes was observed in these mice.

Reduced postprandial suppression of plasma NEFA might be related to the development of insulin resistance through continuous exposure of nonadipose tissues to NEFA (63). This constant exposure reduces insulin-mediated glucose removal, lowers muscle glucose uptake, and stimulates endogenous glucose production (63). We found that WT and ARRDC4KO mice had similar NEFA levels in the fasted state. However, while WT mice showed lowered NEFA levels after refeeding, ARRDC4KO plasma NEFA levels did not decrease much, pointing to a defect in NEFA suppression in these mice in a postprandial state.

MondoA is a critical transcription factor that regulates glucose uptake *via* increased expression of arrestin genes such as *TXNIP* and *ARRDC4* (14). *ARRDC4* and *TXNIP* expressions are increased in human pancreatic beta cells by glucose stimulation through the MondoA transcription factor (14). In this study, *TXNIP* was found to be the most glucose-upregulated gene in EndoC-βH1 cells, followed by *ARRDC4* gene. The fact that both ARRDC4 and TXNIP respond to high glucose stimulation potentially suggests that they have important functions in regulating glucose metabolism.

Our findings suggest that the ARRDC4 protein acts as a regulatory adaptor protein to control an essential membrane receptor protein for maintaining glucose homeostasis. Examples of similar mechanisms also exist in the lower organisms, such as ART proteins of *Saccharomyces cerevisiae*, which regulate membrane abundance of proteins (nutrient transporters) by endocytosis in response to external signals (nutrient abundance) (64). It is possible that these mechanisms continued to evolve in multicellular organisms to maintain homeostasis. Indeed, most of the alpha-arrestin family members play metabolic roles in cells (7). TXNIP plays crucial metabolic roles in multiple organs, such as the heart, liver, muscle, and adipose (65, 66, 67, 68, 69). We also recently found that ARRDC3 directly binds to and regulates insulin receptors in the liver (9). The ARRDC3 protein is also linked to adipocyte function and human obesity (8, 70). Here, we report the major effects of ARRDC4 in the liver; however, ARRDC4 may have important functions in other metabolic organs similar to other arrestin proteins. The functions of ARRDC4 in different metabolic tissues may be revealed by the study of tissue-specific gene deletion models.

## Experimental procedures

### Mice

Global ARRDC4KO mice were previously generated using Arrdc4tm1(KOMP)Vlcg (VG18749) embryonic stem cells, and WT siblings were used as controls (11). All mice were housed at a controlled temperature and a light/dark cycle with free access to a standard chow diet (Prolab Isopro RMH 3000 5P75; LabDiet) and water. All animal studies were performed by the National Institutes of Health (NIH) guidelines and under the approval of Harvard University’s Institutional Animal Care and Use Committee (16-05-271/273). Male mice were used for these metabolic studies. The fasted group was fasted for 16 h, the refed group was refed for 6 h following a 16 h fasting, and the glucagon group was stimulated with glucagon (50 μg/kg) after 16 h of fasting.

### Cell culture

HEPG2 and HEK293 cells were purchased from American Type Culture Collection and cultured according to the American Type Culture Collection protocol.

### Glucose, insulin, PTT, and glucagon challenge

Mice were fasted for 16 h before GTT, glucagon challenge, and PTTs and 4 h before insulin tolerance tests. Fasted mice were injected i.p. with glucose (2 g/kg), pyruvate (2 g/kg), glucagon (20 μg/kg), or insulin (0.75 IU/kg). Blood samples were collected *via* the tail vein, and glucose levels were measured using a handheld glucometer (Contour) at designated time points. For GTTs, insulin levels were measured in plasma samples collected at different time points using ultrasensitive insulin ELISA kit (Alpco). For *arrdc4* mRNA measurements, mice were injected with 2 IU/kg of human insulin (humulin) i.p.

### Biochemistry assays and immunoblots

Glucagon (Sigma–Aldrich), insulin (Alpco), and cAMP (Invitrogen) levels were measured using ELISA kits. In addition, liver triglycerides (Abcam), free fatty acids (Abcam), plasma ketone bodies (Abcam), plasma lactate (Sigma–Aldrich), plasma NEFA (Fujifilm WAKO chemicals), liver glycogen (Sigma–Aldrich), and liver PKA activity (Invitrogen) levels were measured using kits according to the manufacturer’s protocols. Plasma triglycerides, high-density lipoprotein, and cholesterol levels were measured at the BIDMC Small Animal Imaging Facility.

Western blots were performed using PEPCK (Santa Cruz Biotech; catalog no.: H1419), CREB (Cell Signaling; catalog no.: 48H2), P-PKA substrate (Cell Signaling; catalog no.: 100G7E), vinculin (Cell Signaling; catalog no.: 4650), GAPDH (Cell Signaling; catalog no.: 14C10), beta actin (Cell Signaling; catalog no.: D6A8), glucagon receptor (Abcam; catalog no.: ab75240), tGFP (Origene; catalog no.: TA150041), Na/K-ATPase (Cell Signaling; catalog no.: 3010), HIF2 alpha (Abcam; catalog no.: ab109616), DYKDDDDK/FLAG (Cell Signaling; catalog no.: 9A3), p-AKT T308 (Cell Signaling; catalog no.: 244F9), insulin receptor β (Cell Signaling; catalog no.: mAb #3025, p-IGF/IR beta (Cell Signaling; catalog no.: Y1135/1136), GSK beta (Cell Signaling; catalog no.:9315S), P-GSK beta (Cell Signaling; catalog no.: 93365), CRTC2 (Proteintech; catalog no.: 124971AP), PYGL (Abcam; catalog no.: 190243), p-PYGL (Invitrogen; catalog no.: PA5-114628), PGC-1α (EMD Millipore; catalog no.: AB3242), GS (Cell Signaling; catalog no.: 3893S), phosphorylated state of GS (Cell Signaling; catalog no.: 3891S) antibodies and secondary horseradish peroxidase–conjugated antibodies from Bio-Rad. Plasma membrane proteins were isolated using the Plasma Membrane isolation kit from Abcam that removes all cellular membrane proteins (catalog no.: ab65400).

For quantitative RT–PCR, RNA was extracted from homogenized liver samples by the TRIzol (Life Sciences) method according to the manufacturer’s protocol. The complementary DNA was synthesized from 2 μg of total RNA by use of the High-Capacity cDNA synthesis kit (Applied Biosystems), and reactions were performed using the Taqman gene expression assays (Applied Biosystems). The following probes were used: *Tbp*: Mm00446971_m1; *Arrdc4*: Mm00508442_m1; *G6Pc*: (Mm00839363_m1); and *Creb1*: Mm00501607_m1). Expression of the target genes was normalized to *Tbp* housekeeping gene expression and control groups.

### Coimmunoprecipitation and internalization

HEPG2 cells were transfected with human ARRDC4-C-Myc/FLAG (Origene; catalog no.: RC205534), human glucagon receptor-GFP (Origene; catalog no.: RG211179), and pCMV6 control plasmids (5 μg) using Lipofectamine 3000 (Thermo Fisher) according to the manufacturer’s instructions. Coimmunoprecipitation assays were performed as described (9). Briefly prewashed beads that were conjugated with anti-GFP (MBL) or anti-FLAG magnetic antibodies were incubated together with 500 μg of lysates from each sample (in 50 mM Tris–HCl, 500 mM NaCl, 1 mM EDTA, 1% Triton X-100, pH 7.4 + protease inhibitor) for 1 h at 4 °C. The samples were washed 3× with washing buffer (50 mM Tris, 150 mM NaCl, 1% Triton X-100, pH 7.4) using a magnet stand. Precipitated proteins were dissociated from beads by competitive elution with either FLAG or GFP peptides (0.15 mg/ml) by shaking at 4 °C for 40 min. Supernatants were used for immunoblotting assays using FLAG and GFP antibodies. Samples were stimulated with glucagon (200 nm) for 15 min.

For internalization experiments, HEK293 cells were plated in 10 cm dishes in duplicates and transfected with either an empty or hARRDC4 plasmid + human glucagon receptor plasmids. Two days later, the cells were starved for 1 h in serum-free media and stimulated with glucagon (500 nM) for 0, 5, 10, 15, 60, and 120 min, and cell surface proteins were immediately isolated using a commercial kit (Abcam). Glucagon receptor levels were measured using a glucagon receptor ELISA kit according to the manufacturer’s protocol (Mybiosource) and normalized to total protein levels. Data were normalized to basal percent levels.

### Hyperinsulinemic–euglycemic clamps

Whole-body fat and lean masses were noninvasively measured using proton magnetic resonance spectroscopy (Echo Medical Systems). Hyperinsulinemic–euglycemic clamp experiments were conducted at the National Mouse Metabolic Phenotyping Center at UMass Medical School. Briefly, survival surgery was performed 5 to 6 days before clamp experiments to establish a catheter in the jugular vein. A 2 h hyperinsulinemic–euglycemic clamp was conducted in overnight-fasted awake mice with a primed (150 mU/kg) and continuous (2.5 mU/kg/min) infusion of human insulin. Glucose concentrations during the clamps were analyzed using Analox GM9 Analyzer using plasma from the tail vein (Analox Instruments). Glucose (20% dextrose) was infused at variable rates during the clamps to maintain euglycemia. Whole-body glucose turnover was measured with a continuous infusion of [3-^3^H] (0.1 mCi/min) glucose during the clamp. A bolus of 2-deoxy-d-[1–14 C] glucose, (2-[14 C]DG) (10 m Ci), was administered 75 min after the start of the clamp. Hepatic glucose production was determined by subtracting the glucose infusion rate from the whole-body glucose turnover. Whole-body glycogen plus lipid synthesis is calculated as the difference between whole-body glucose turnover and whole-body glycolysis. At the end of the clamps, mice were anesthetized, and individual tissues were taken for biochemical analysis. Plasma concentrations of [3-^3^H]glucose, 2-[^14^C]DG, and ^3^H_2_O were determined following deproteinization of plasma samples using ZnSO_4_ and BaOH. Calculations were carried out as described previously (18).

### Confocal microscopy

HEPG2 cells seeded on 35 mm glass bottom dishes were transfected with an mCherry-hARRDC4 (modified from Origene h-ARRDC4 plasmid; catalog no.: RC205534) and GFP-hGCGR (Origene; catalog no.: RG211179) plasmid and imaged live in a Zeiss LSM880 confocal microscope with a 63× Plan-Apochromat 1.4 numerical aperture oil objective. GCGR-GFP was excited with a 488 nm argon laser line with emission collected from 493 to 571 nm, and ARRDC4-mCherry was excited with a 561DPSS laser line with emission data collected from 571 to 754 nm. Excitation was done sequentially to minimize crosstalk between the two channels. Cells were chosen for imaging based on morphology. Nearby untransfected cells in the field of view provided controls to confirm the signal was not coming from autofluorescence.

### Statistical analysis

Data are expressed as means ± SE. The significance of the difference in mean values was determined using one-way and two-way ANOVA with multiple comparison post hoc tests and Student's *t* test where applicable. The statistical significance was set at a *p* value of <0.05.

## Data availability

The background data may be requested from the author.

## Supporting information

This article contains supporting information.

## Conflict of interest

R. T. L. is a cofounder, scientific advisory board member, and private equity holder of Elevian, Inc. R. T. L. is a member of the scientific advisory board of Revidia Therapeutics, Inc, and the laboratory receives research support from BlueRock Therapeutics. J. K. K. is a scientific advisor of Elevian, Inc. All other authors declare that they have no conflicts of interest with the contents of this article.
