# Supplementary material for: An insulin-regulated arrestin domain protein controls hepatic glucagon action
Source: J Biol Chem. 2023 Jul 13;299(8):105045. doi: 10.1016/j.jbc.2023.105045 (PMC10413355; doi:10.1016/j.jbc.2023.105045)
Supplement: Supplementary figures [file mmc1.pdf]

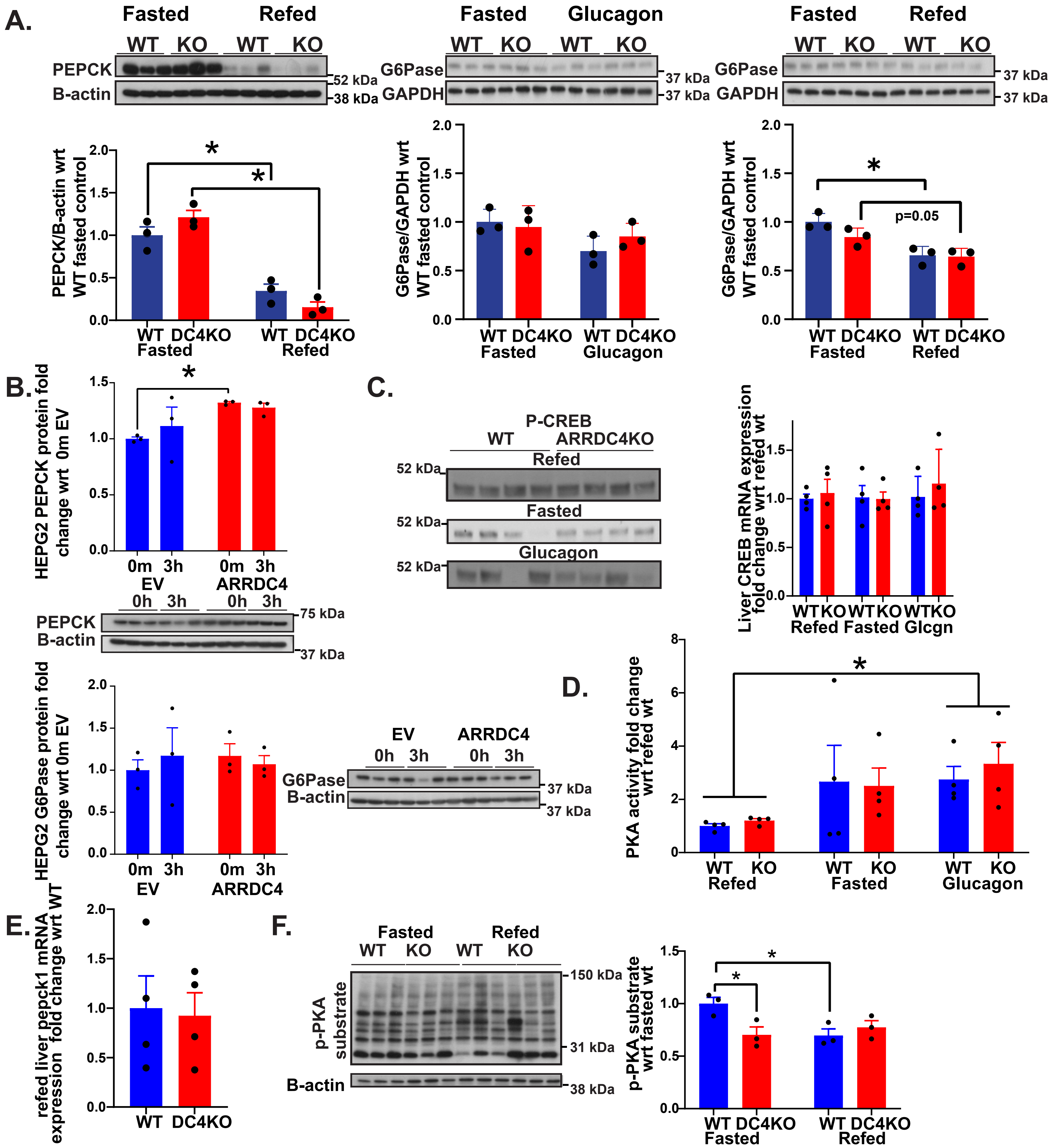

Supplementary Figure 1. (A) PEPCK and G6Pase protein levels in 16 hr-fasted+6-hr-refed, 16-hr-fasted and 16-hr-fasted+ glucagon-stimulated WT and ARRDC4KO mouse livers (n=3), (B) G6Pase and PEPCK protein levels in HEPG2 cells that are transfected with ARRDC4 or empty vector and stimulated with insulin for 3 hrs (n=3), (C) p-CREB (n=3) and *Creb* mRNA (n=4) levels in refed, fasted and glucagon-stimulated mice livers, (blotted on the same membrane as total CREB), (D) Hepatic PKA activity levels following 16-hr fasting+ 6-hr refeeding, 16-hr fasting or 16-hr fasting+glucagon stimulation in WT and ARRDC4KO mice (n=4), (E) *Pepck1* mRNA levels in refed WT and ARRDC4KO mouse livers (n=4), (F) P-PKA substrate levels in the livers of 16-hr-fasted and 16-hr-fasted + 6-hr-refed WT and ARRDC4KO mice (n=3),($p<0.05$, values are mean±SEM, two-way ANOVA and unpaired two-tailed student t-test).

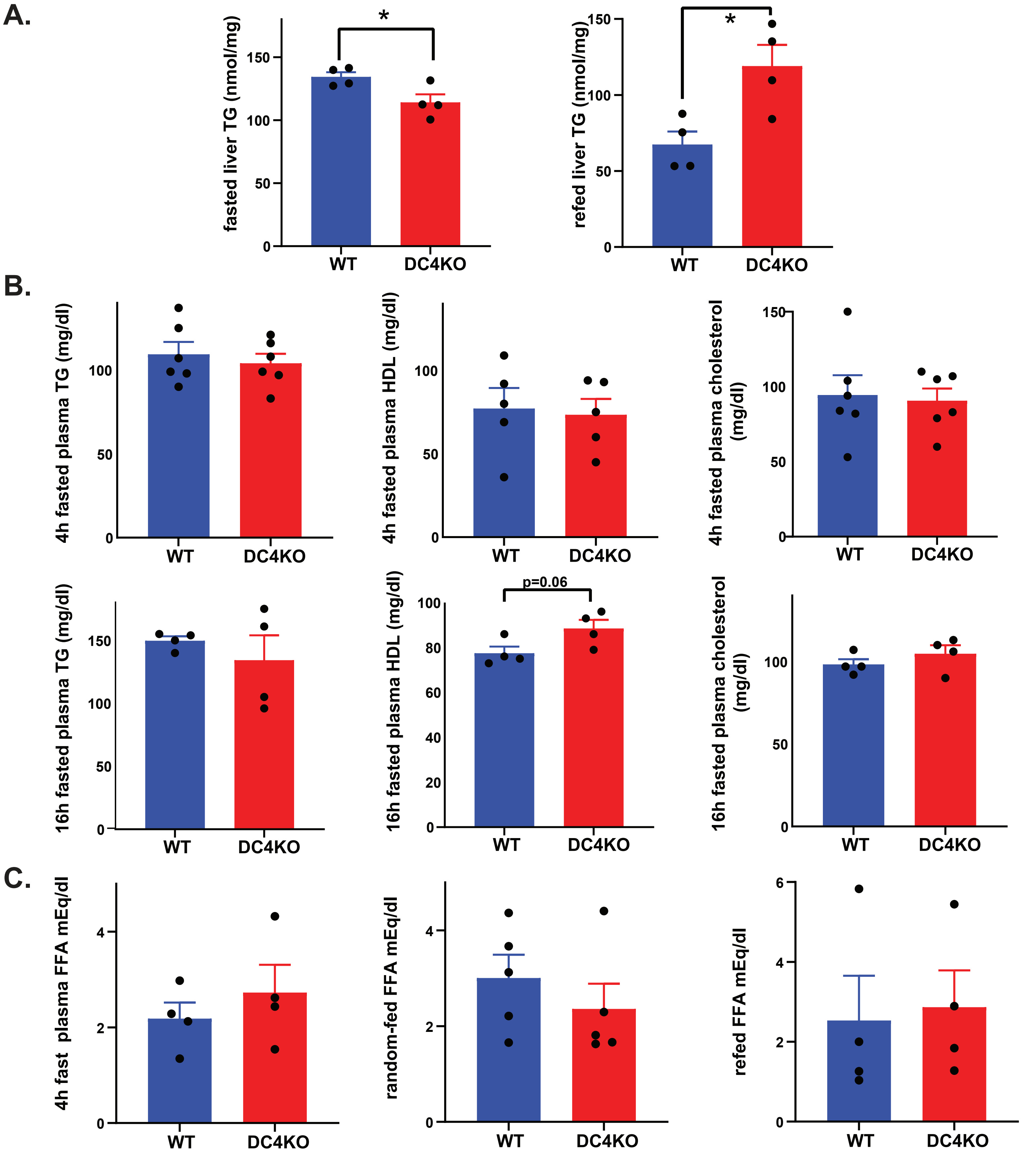

Supplementary Figure 2. (A) Hepatic triglyceride levels in 16-hr-fasted and 16-hr-fasted+ 6-hr refed WT and ARRDC4KO mice (n=4), (B) 4- and 16-hour fasted plasma triglyceride, HDL and cholesterol levels in WT and ARRDC4KO mice (n=4), (C) Plasma free fatty acid levels in 4-hr fasted, random-fed and 16-hr-fasted+ 6-hr refed adult male WT and ARRDC4KO mice (n=4)

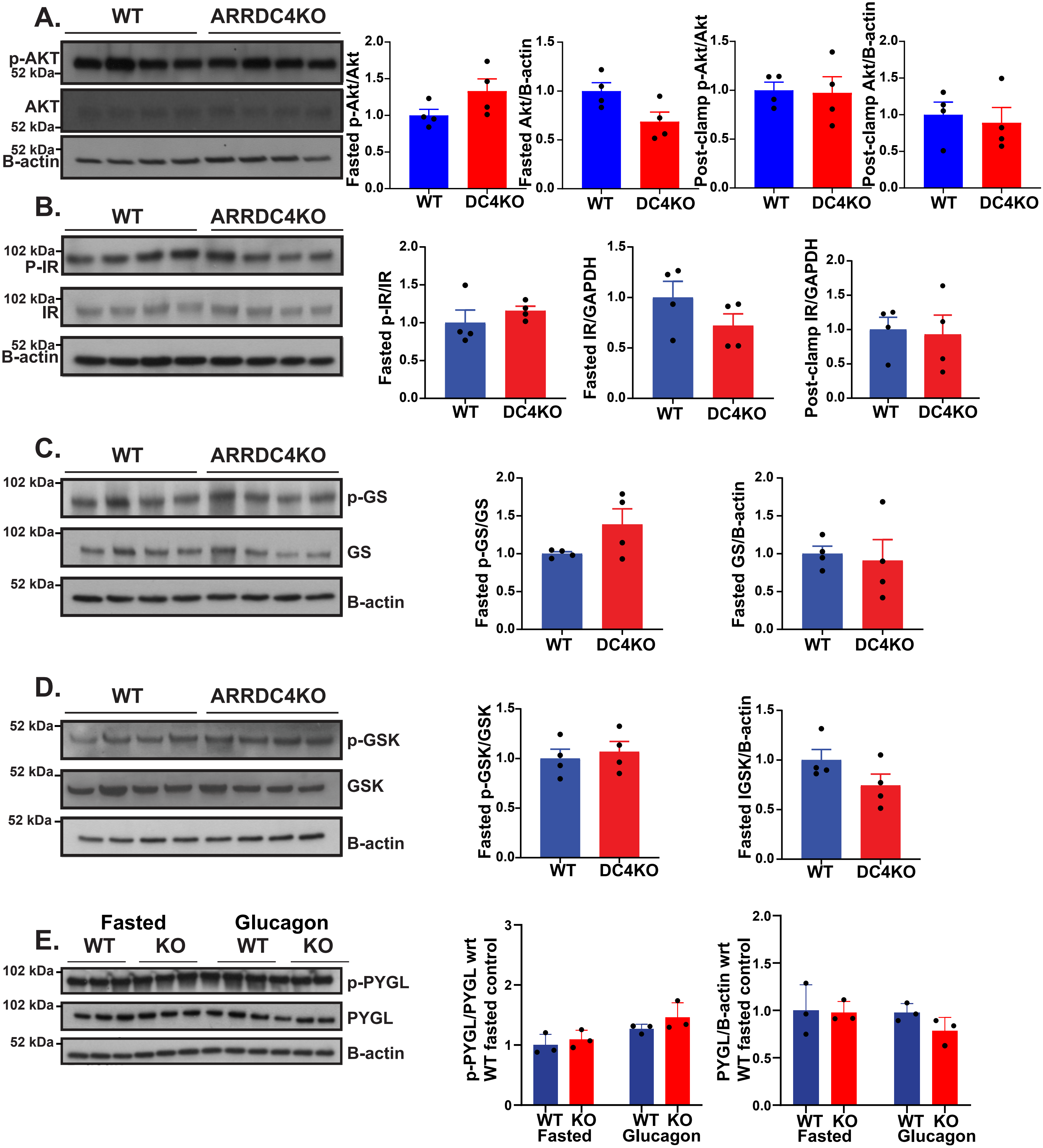

Supplementary Figure 3. (A) p-Akt and total Akt protein levels at 16-hr fasted and post-clamp state (n=4), (B) p-Insulin receptor and total insulin receptor levels at overnight fasted and post-clamp state (n=4), (C) p-glycogen synthase and total glycogen synthase levels, (D) p-glycogen synthase kinase and total glycogen synthase kinase levels in 16-hour fasted state in livers of WT and ARRDC4KO mice (n=4), (E) p-PYGL and total PYGL levels in 16-hour fasted and 16-hour fasted+glucagon stimulated state in livers of WT and ARRDC4KO mice (n=3) ($p<0.05$, values are mean ±SEM, and two-tailed student t-test).
